# Usefulness of dermoscopy in the evaluation of patch test reactions^☆^^☆☆^

**DOI:** 10.1016/j.abd.2021.04.006

**Published:** 2021-10-08

**Authors:** Kenselyn Oppermann, Cristiane Almeida Soares Cattani, Renan Rangel Bonamigo

**Affiliations:** a Postgraduate Program in Pathology, Universidade Federal de Ciências da Saúde de Porto Alegre, Porto Alegre, RS, Brazil; b Department of Health, Ambulatório de Dermatologia Sanitária de Porto Alegre, Porto Alegre, RS, Brazil; c Faculty of Medicine, Universidade Federal do Rio Grande do Sul, Porto Alegre, RS, Brazil

**Keywords:** Allergic contact, dermatitis, Dermoscopy, Patch tests

## Abstract

**Background:**

Despite being widely used in different areas of dermatology, there have been few studies evaluating the benefit of dermoscopy in the interpretation of patch tests, especially in weak and doubtful reactions.

**Objectives:**

To evaluate the role of dermoscopy in the interpretation of patch tests and describe the main findings of the reactions.

**Method:**

Prospective study, carried out in dermatology reference centers in southern Brazil, which evaluated the final results of patch tests analyzed with the aid of dermoscopy.

**Results:**

77 patients and 160 reactions were included. The most prevalent substances were nickel sulphate (23.8%), kathon CG (9.4%), and perfume mix (8.8%). The main dermoscopic findings were reaction area​​ greater than half of the chamber site (90%), homogeneous erythema (86.9%), vesicles (30%), crusts (21.3%), perifollicular erythema (35%), pore reaction (19.4%) and pustules (8.8%). Dermoscopy was found to facilitate the definition of erythema in reactions on black skin and when due to substances with deposition of pigment. Of the 64 weak or doubtful reactions, 36 (56.25%) showed a change in the final result after dermoscopy evaluation; of the 36 doubtful reactions, 33 (91.6%) showed a change in the final result after dermoscopy evaluation (p < 0.001).

**Study limitations:**

The probable limitation of the study is its sample size. Though certain significance levels have been reached, other possible relationships may not have been observed.

**Conclusion:**

Dermoscopy improves significantly the interpretation of patch tests, especially in weak and doubtful reactions.

## Introduction

Dermoscopy is a non-invasive, safe, and practical method that is widely used in the diagnosis and follow-up of several dermatoses, especially melanocytic lesions.^1^, ^2^, ^3^, ^4^ Despite the large number of studies describing the importance of dermoscopy in general dermatology, there have been few studies evaluating the usefulness of this complimentary exam in the interpretation of patch tests.^5^, ^6^, ^7^, ^8^

For the interpretation of patch tests, even when following internationally accepted reading criteria, there are difficulties for the conclusion of certain cases of suspected Allergic Contact Dermatitis (ACD), especially in differentiating weak and doubtful reactions.^9^, ^10^

Establishing the role that dermoscopy can play in improving the interpretation of patch tests was the objective of the present study, as well as describing the main dermoscopic findings of the reactions.

## Methods

A prospective study was carried out with patients referred for patch tests in two reference Dermatology Services in southern Brazil, Ambulatório de Dermatologia Sanitária do Rio Grande do Sul and Hospital de Clinicas de Porto Alegre, between November 2018 and December 2019. The study was approved by the respective Research Ethics Committees, under numbers 2,952,421, 3,032,971, 2,990,263, and all participants signed the free and informed consent form, in accordance with the Declaration of Helsinki and the National Health Council Resolution of 2012.

Dermoscopy was performed for the interpretation of tests routinely done in the Services, which used – according to the necessary indication – the Brazilian standard set of tests consisting of 30 substances or a pediatric set, consisting of 20 substances, and a cosmetic set, consisting of 10 substances (all test sets manufactured by Asacpharma®). All patients with positive or doubtful reactions at the 96-h reading (second reading or final reading) were included for dermoscopic evaluation. Patients with negative 96-h readings were excluded.

The assessment (reading) of the tests after 96 hours was interpreted with the naked eye (by an expert dermatologist – R.R.B. and a researcher dermatologist – K.O.), according to the criteria of the International Contact Dermatitis Research Group (ICDRG), as follows: doubtful reaction – some erythema (?); mild reaction – definite erythema, mild infiltration and occasional papules (1+); strong reaction – definite erythema, infiltration, occasional papules and vesicles (2+); very strong reaction – intense erythema, infiltration and coalescing vesicles (3+).^11^, ^12^, ^13^, ^14^, ^15^ Doubtful or positive reactions within 48 hours, which became negative at the naked eye reading (absence of erythema) within 96 hours, were classified as irritant reactions.

The interpretation of the patch tests through dermoscopy was performed with the aid of a Dermlite 4 dermatoscope (×10 magnification), and the images of the reactions were recorded using Handyscope FotoFinder® (×20 magnification) and FotoFinder® (×40 magnification), with a thick interface gel layer and minimal pressure. Dermoscopic images were obtained by the researcher dermatologist (K.O.) and evaluated by R.R.B. and K.O.

The results obtained at the dermoscopic evaluation involved the following variables: - Total area of ​​skin involvement at the chamber site: up to 50% or greater than 50%; - homogeneous erythema: diffuse erythema over most of the chamber area; - Perifollicular erythema: erythema found essentially around the follicular ostia; - Papules – lighter areas simulating a veil, poorly circumscribed; - Vesicles/blisters – whitish circles of different sizes that resemble soap bubbles; - Pustules: yellowish circles; - Crusts: solid and adherent material from fluid drying; - Pore reaction (or follicular obstruction): follicular reaction pattern, which may be brownish pigment or formation of crusts in the follicular ostia; - Follicular accentuation: multiple small grayish-white dots of the same size.

The vascular alterations were classified as: - Punctiform vessels: small dots; - Linear; - Polymorphic: when they do not follow a pattern and appear as more than one form; - Petechiae: purplish color that does not disappear on glass pressure. These criteria were chosen from previously published studies and adapted according to the authors' experience.^7^, ^8^, ^16^, ^17^

The main dermoscopic characteristics of allergic reactions include homogeneous and diffuse erythema, affecting or not the follicular area, papules and fluid accumulation. There is the possibility of a set of accessory and secondary changes such as crusts, pore reaction, and follicular accentuation, but these findings alone, without the formation of homogeneous erythema (with or without papules and fluid accumulation), do not constitute allergic-type reactions. Vascular alterations are also accessory findings to be better explored, as in the present study.

Irritant reactions were not specifically considered in the analysis, as the objective was to evaluate reactions that remained with erythema for 96 hours, but it is important to mention that the dermoscopic characteristics of irritant reactions are described as pore reaction and/or perifollicular reaction, in the absence of homogeneous basal erythema. As with allergic reactions, there is no clear pattern of vascular changes specific for irritant reactions.^7^, ^8^, ^17^ In this study, results with irritant reactions were considered negative for ACD after dermoscopy.

The collected information was entered into a Microsoft Excel program database, processed and analyzed using the SPSS software, version 25. Data were presented as frequencies and percentages, and age as mean and standard deviation. Associations were verified using the Chi-Square test or Fisher's exact test, as appropriate. The ‘before’ and ‘after’ results were compared using McNemar's test. Results were considered significant with a p-value <0.05.

## Results

Seventy-seven patients were included, with a total of 160 reactions. It was observed that most of the sample consisted of women (77.9%), with a wide age range and phototype variability, and a personal history of atopy in 15.6% of the patients; most were tested using the standard set of tests (76.6%).

The most prevalent substances were nickel sulfate (23.8%), the isothiazolinone group – “Kathon CG” (9.4%), and the perfume group – “Perfume-mix” (8.8%). The general characteristics of patients submitted to patch test reading with dermoscopy are described in Table 1.

**Table 1:** Characteristics of the studied sample (X/Y, 2018–2019).

	Result
**Patients** (n)	77
**Age (years)** mean (minimum-maximum)	41.3 (3–75)
**Sex,** n (%)	
Female	60 (77.9)
Male	17 (22.1)
**Phototype,** n (%)	
I	2 (2.6)
II	21 (27.3)
III	16 (20.8)
IV	23 (29.9)
V	12 (15.6)
VI	3 (3.9)
**History of atopy,** n (%)	
Personal	12 (15.6)
**Set of tests used,** n (%)	
Standard	59 (76.6)
Pediatric	16 (20.8)
Cosmetic	2 (2.6)
**Reactions** (n)	160
**More prevalent substances,** n (%)	
Nickel sulfate	38 (23.8)
Kathon CG (isothiazolinone)	15 (9.4)
Perfume mix	14 (8.8)
Paraphenylenediamine	9 (5.6)
Neomycin	8 (5.0)
Thimerosal	7 (4.4)

**Figure 1:**
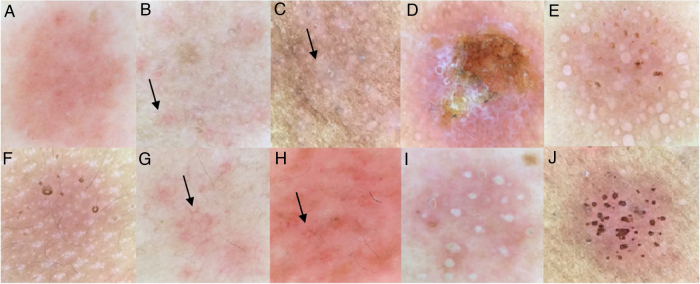
Dermoscopy findings of the patch tests: (a), Homogeneous erythema; (b), Perifollicular erythema (arrow); (c), Papules (arrow); (d), Crusts; (e), Vesicles; (f), Follicular accentuation (atopic patient); (g), Linear vessels; (h), Petechiae (arrow); (i), Pustules; (j), Pore reaction pattern.

The main dermoscopic findings of patients with ACD were: reaction area​​ greater than half of the chamber site (90%), homogeneous erythema (86.9%), vesicles (30%), crusts (21.3%), perifollicular erythema (35%), pustules (8.8%), pore reaction (19.4%) and vascular alterations (21.3%) (Table 2, Fig. 1). The substances that most often showed a pore reaction pattern were nickel sulfate (39.5%) and cobalt (28.6%).

**Table 2:** Dermoscopic findings of patch test reactions at the 96-h readings.

Dermoscopic finding	Doubtful Reaction (n = 3)	(+) Reaction (n = 44)	(++) Reaction (n = 49)	(+++) Reaction (n = 47)
Reaction area >50%	2 (66.7%)	40 (90.9%)	48 (97.9%)	47 (100%)
Homogeneous erythema	2 (66.7%)	41 (93.1%)	47 (95.9%)	47 (100%)
Perifollicular erythema	3 (100%)	17 (38.6%)	15 (30.6%)	8 (17.0%)
Papules	1 (33.3%)	22 (50.0%)	42 (85.7%)	36 (76.5%)
Vesicles/bubbles	0 (0.0%)	0 (0.0%)	19 (38.7%)	29 (61.7%)
Pustules	0 (0.0%)	0 (0.0%)	3 (6.1%)	9 (19.1%)
Crusts	0 (0.0%)	1 (2.2%)	6 (12.2%)	26 (55.3%)
Follicular obstruction	0 (0.0%)	5 (11.3%)	6 (12.2%)	13 (27.6%)
Follicular accentuation	0 (0.0%)	5 (11.3%)	4 (8.1%)	3 (6.3%)
Vascular alterations				
Punctiform vessels	0 (0.0%)	0 (0.0%)	5 (10.2%)	5 (10.6%)
Linear vessels	0 (0.0%)	3 (6.8%)	4 (8.1%)	0 (0.0%)
Petechiae	0 (0.0%)	0 (0.0%)	2 (4.0%)	5 (10.6%)
Polymorphic vessels	0 (0.0%)	1 (2.2%)	3 (6.1%)	1 (2.1%)
Uncharacterized vessels	3 (100%)	40 (90.9%)	35 (71.4%)	36 (76.5%)

**Figure 2:**
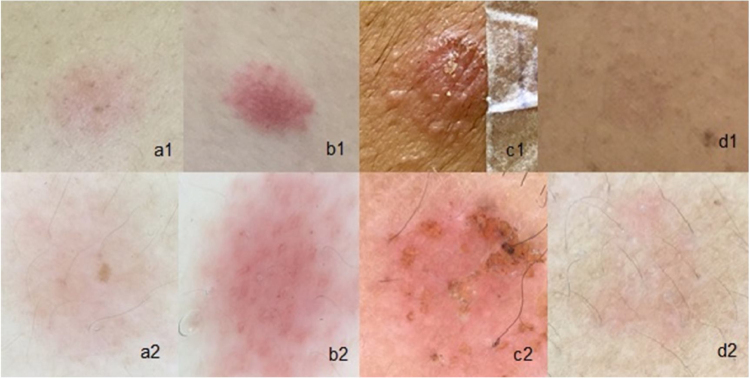
Patch tests: (a1), Weak reaction (1+) with the naked eye; (a2), Weak (1+) with dermoscopy; (b1), Strong (2+) with the naked eye; (b2), Strong (2+) with dermoscopy; (c1), Very strong (3+) with the naked eye; (c2), Very strong (3+) with dermoscopy; (d1), Doubtful reaction observed with the naked eye; (d2), 1+ reaction with dermoscopy.

The morphological vascular alterations comprised punctiform, linear, polymorphic, petechial, and uncharacterized vessels. In 21.3% of the cases, it was possible to characterize the predominant vascular morphology, namely: 6.3% punctiform vessels; 5% linear vessels; 3.1% polymorphic, and 6.9% petechiae. In the other cases (almost 79%) there was a predominance of basal erythema, with no differentiation in vascular morphology. The images of the patch test reactions, according to the ICDRG, observed with the naked eye and with dermoscopy, are shown in Figure 1, Figure 2. Moreover, it is important to emphasize the importance of dermoscopy when defining erythema in doubtful reactions.

The reactions were considered negative on dermoscopy when they did not have an area of ​​diffuse erythema, even if isolated perifollicular erythema and follicular crusts were present. Doubtful reactions on dermoscopy were considered those where the doubt about the existence of any non-follicular area of ​​erythema remained (similarly to the evaluation with the naked eye).

Of the 160 reactions studied, 28 (17.5%) were weak, 49 (30.6%) were strong, 47 (29.3%) were very strong, and 36 (22.5%) were doubtful. When evaluating the 36 doubtful reactions observed with the naked eye, 33 (91.6%) showed a change in the final result after dermoscopy (p < 0.001): 17 were reclassified as negative reactions and 16 as weakly positive reactions (+).

Given the relatively small number of patients with a personal history of atopy, it was not possible to correlate this variable to a specific pattern of dermatological findings in the final reading of the patch tests.

## Discussion

Dermoscopy seems to reveal important changes when used in the interpretation of patch tests in suspected ACD.^7^, ^8^, ^17^ Corazza et al. primarily assessed the differences between allergic and irritant reactions and observed that erythema is the most important one when differentiating between irritant and allergic reactions.^8^

The main objective of this study was to describe the dermoscopic findings observed in the final evaluation of patch test reactions, in patients with suspected ACD.

In tests with strong (2+) and very strong (3+) reactions observed with the naked eye, dermoscopy wasn’t decisive for the diagnosis, as in these cases the clinical features are evident and can be complemented with palpation. In doubtful and weak reactions, it can be useful in improving the interpretation of results. Of the 160 reactions studied, 28 (17.5%) were weak and 36 (22.5%) were doubtful before dermoscopy.

**Figure 3:**
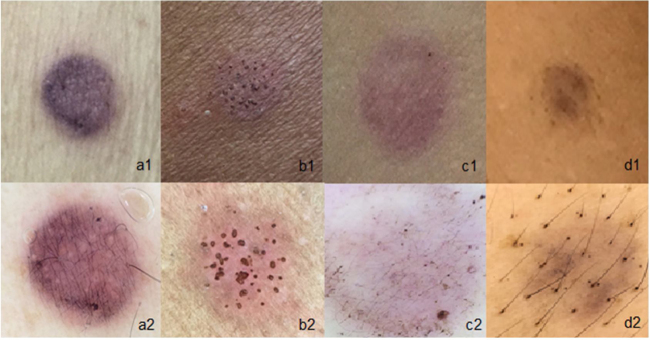
Usefulness of dermoscopy in patch tests that contain pigment: (a1), Naked-eye paraphenylenediamine test, with doubtful reaction; (a2), Paraphenylenediamine test with weak reaction (1+); (b1), Patient phototype V with reaction to nickel observed with the naked eye, with mild erythema; (b2), The same patient with dermoscopy showing well-defined erythema and pore reaction; (c1), Reaction to Disperse Blue observed with the naked eye, with doubtful reaction; (c2), Reaction to Disperse Blue with dermoscopy showing only pigment deposition without erythema (negative reaction); (d1 and d2), Test with Paraphenylenediamine, observed with the naked eye and with dermoscopy, showing only pigment deposition without erythema (negative reaction).

Dermoscopy allowed a better definition of erythema, leading to the reclassification of doubtful reactions in 91.6% of the cases (p < 0.001%), reaching a positivity in 44.4% of reactions initially classified as doubtful, and 17 reactions were reclassified as negative. After dermoscopy, only three reactions remained doubtful (Table 3); these findings were statistically significant. Obviously, weak (+) reactions may or may not be considered clinically relevant, as some reactions are unrelated to the patient's clinical condition. Dermoscopy alone is a method to disclose a possible diagnosis that could go unnoticed; therefore, the correlation between test results and clinical investigation (history and physical examination) remains crucial. Dermoscopy seems to provide a better assessment of tests in patients with high phototypes and is helpful in the assessment of substances with pigment deposition, especially in revealing homogeneous erythema (Fig. 3).

**Table 3:** Reactions before and after dermoscopy.

Results of reactions in 96h^a^	Naked eye, n (%)	Dermoscopy, n (%)	p
Weak	28 (17.5%)	44 (27.5%)	
Strong	49 (30.6%)	49 (30.6%)	
Very strong	47 (29.4%)	47 (29.4%)	
Doubtful	36 (22.5%)	3 (1.9%)	<0.001
Negative/irritant^b^	-	17 (10.6%)	

a According to the International Contact Dermatitis Research Group.

b Negative/irritant reactions to the naked eye were not included, but were included after revealed in the dermoscopy.

Unlike the study by Corazza et al., the present study did not show vesicles in weak reactions, but it was a very present finding in strong and very strong reactions, and they were characterized by whitish circles of different sizes, resembling “soap bubbles”, as described in the literature.^8^ Regarding the characteristics of the vascular pattern in allergic reactions, there was a predominance of diffuse erythema (79%) of different degrees.^8^, ^16^

## Conclusion

This study demonstrates that dermoscopy is very useful in the interpretation of weak reactions and even more in doubtful reactions; it also provides a better definition of erythema in reactions in patients with phototypes IV and V and in reactions due to substances with pigment deposition.

## Financial support

None declared.

## Authors’ contributions

Kenselyn Oppermann: Statistical analysis; approval of the final version of the manuscript; design and planning of the study; drafting and editing of the manuscript; collection, analysis and interpretation of data; intellectual participation in propaedeutic and/or therapeutic conduct of the studied cases; critical review of the literature; critical review of the manuscript.

Cristiane Almeida Soares Cattani: Approval of the final version of the manuscript; effective participation in research orientation; intellectual participation in propaedeutic and/or therapeutic conduct of studied cases; critical review of the literature; critical review of the manuscript.

Renan Rangel Bonamigo: Statistical analysis; approval of the final version of the manuscript; design and planning of the study; drafting and editing of the manuscript; collection, analysis, and interpretation of data; effective participation in research orientation; intellectual participation in propaedeutic and/or therapeutic conduct of the studied cases; critical review of the literature; critical review of the manuscript.

## Conflicts of interest

None declared.
